# Understanding the Burden of Mental Illness Induced by Workplace Mobbing: A Scoping Review

**DOI:** 10.62641/aep.v53i5.1930

**Published:** 2025-10-05

**Authors:** Hernan G. Rincon-Hoyos, Roger Figueroa-Paz, María Mercedes Cardozo-Rengifo, Daniela Gil-González, Juan F. Zúñiga-Martinez, Oriana Arias-Valderrama, Andrés Gempeler

**Affiliations:** ^1^Servicio de Psiquiatría, Departamento de Medicina Interna, Fundación Valle del Lili, 760032 Cali, Colombia; ^2^Facultad de Ciencias de la Salud, Universidad Icesi, 760031 Cali, Colombia; ^3^Centro de Investigaciones Clínicas, Fundación Valle del Lili, 760032 Cali, Colombia

**Keywords:** workplace violence, mobbing, occupational stress, cost of illness, productivity, absenteeism

## Abstract

**Background:**

Workplace mobbing affects approximately 20% of workers worldwide, yet about 70% of victims do not report it, limiting the full understanding of its true impact. While previous research has established its association with mental health disorders, the broader burden—including burden of disease (BOD), cost of illness (COI), and productivity loss (PL)—remains underexplored. This scoping review aims to address this gap by mapping the existing literature on the BOD and economic costs associated with mobbing-related mental health disorders.

**Methods:**

We conducted a scoping review following the Preferred Reporting Items for Systematic Reviews and Meta-Analyses extension for Scoping Reviews (PRISMA-ScR) guidelines. A systematic search in National Library of Medicine’s bibliographic database (MEDLINE), Latin American and Caribbean Literature in Health Sciences (LILACS), Embase, Scopus, and PsycInfo databases (until June 30, 2021) identified primary studies and reviews assessing BOD, COI, or PL in adults exposed to workplace mobbing. Articles were screened independently by two reviewers in two phases (title/abstract and full-text review). Data extraction focused on study characteristics and key findings, which were categorized into predefined thematic domains.

**Results:**

Fourteen studies published between 2008 and 2020 met the selection criteria (71.4% primary studies, 28.6% reviews). The definition of mobbing varied across studies, and frequently, different terms were used interchangeably. None of the included studies quantified disease burden using standard metrics such as disability-adjusted life years (DALYs). Instead, PL was assessed indirectly through absenteeism, presenteeism, and work performance assessments.

**Conclusions:**

Mobbing is a significant occupational health issue with substantial mental health implications, yet research on its economic and disease burden remains limited. The heterogeneity in definitions and methodologies across studies hampers comparability and synthesis. Future research should adopt standardized definitions and employ robust burden-of-disease frameworks, such as DALYs and Quality-Adjusted Life Years (QALYs), to better quantify the impact of mobbing on mental health and work productivity.

## Introduction

Workplace harassment, commonly referred to as mobbing, is a form of deliberate and sustained hostile behavior directed at an individual within a work environment. This conduct may involve threats, intimidation, humiliation, or other aggressive actions that cause physical, psychological, or economic harm [1]. While previous research has focused on the direct links between mobbing and the development of mental health disorders, there is a significant gap in the literature regarding the broader consequences, specifically the burden and cost of disease related to mobbing-induced mental illness [2, 3, 4]. Although mental health outcomes, such as depression and anxiety, are often studied in relation to mobbing, their economic impact on productivity, disability, and overall disease burden remains underexplored. Understanding these effects is critical, not only for the well-being of individuals but also for the broader implications for organizations and society [5].

Estimates suggest that approximately one in five individuals globally experiences mobbing on a weekly basis [6, 7], with even higher rates reported in high-income countries [6]. The Americas have the highest prevalence, with 43.3% of individuals affected, followed by Africa (25.7%), Europe and Central Asia (25.5%), and Asia-Pacific (19.2%). In Latin America, studies from Mexico and Chile indicate a prevalence range of 14% to 76.5%. These statistics highlight the widespread nature of mobbing, yet its economic and health-related consequences are not fully quantified or understood [8, 9, 10, 11, 12, 13].

It has been shown that work related depression comorbid with physical disease severely affects individuals’ quality of life and well-being, the resulting consequences are often seen in increased absenteeism, decreased work performance, and a decline in overall productivity [14]. In the context of mobbing-induced mental illness, however, accurately measuring the burden and cost is complex. Many existing frameworks for calculating the burden of disease (BOD) fail to account for the full impact on an individual’s overall well-being, as they focus on loss of health without considering the broader social and economic implications of mental health problems. This limitation is especially problematic for mental health disorders, where comorbid conditions are common, and the cumulative effect on a person’s health and productivity is often underestimated [5].

Furthermore, the typical models for estimating the cost of illness (COI) often assume that comorbid conditions are independent of each other, an assumption that does not hold true in the case of mental health disorders, which frequently co-occur with physical health problems. The interrelationship between mental and physical health conditions exacerbates the challenge of accurately estimating the burden and economic costs associated with mobbing [5]. Therefore, a focused investigation into the burden and cost of mental illness resulting from mobbing will provide invaluable insights for both healthcare and occupational health sectors, informing strategies to mitigate these impacts.

This scoping review aims to address this gap by mapping the existing literature on the BOD and cost of mental health disorders secondary to mobbing. The findings will lay the groundwork for future studies that seek to develop more accurate and comprehensive methods for assessing the impact of mobbing on both individual health outcomes and societal productivity.

## Methods

### Design

We conducted a scoping review according to the methodological framework proposed by Arksey and O’Malley [15]. Additionally, we followed the conducting and reporting recommendations of the Joana Briggs Institute manual [16] and the Preferred Reporting Items for Systematic Reviews and Meta-Analyses extension for Scoping Reviews (PRISMA-ScR) statement [17].

### Search Strategy

We searched MEDLINE, Embase, Psycinfo, Scopus and LILACS/Scielo databases on June 30, 2021. Both free and controlled terms were combined with boolean operators, with no language or time restrictions. Box 1 contains the generic search strategy.

**Box 1.** **Search strategy**.

1. Non-sexual Harassment **AND** Workplace
2. Occupational Stress **AND** Bullying
3. Occupational Stress **AND** Workplace
4. Bullying **AND** Workplace
5. Mobbing **OR** 1 **OR** 2 **OR** 3 **OR** 4
6. Burden of disease **OR** Cost of illness **OR** Mental Health **OR** Mental Disorders **OR** Depression **OR** Anxiety **OR** Post-traumatic stress disorder **OR** Sleep disorders
7. 5 **AND** 6

### Selection Criteria 

The following inclusion and exclusion criteria were applied.

#### Inclusion Criteria

a. Original research papers and review papers (i.e., systematic and non-systematic literature reviews) with all descriptive and analytical study designs.

b. Publications written in English, Spanish or German (languages spoken by the authors).

c. Articles reporting data on adults exposed to mobbing that described or assessed its BOD alone or in association with mental health, in terms of BOD, COI or productivity loss (PL) (see definitions).

#### Exclusion Criteria

Opinion articles, editorials, case reports, and book chapters.

### Definitions

We considered the terms “mobbing”, “workplace harassment”, “workplace bullying” and similar as synonymous.

#### BOD

Quantifies the total impact of a disease, injury or risk factor on a given population in terms of mortality, morbidity and disability. Studies that used/or reported health utility measures such as DALYs, QALYs, Years Lived with Disability (YLDs), Years of Life Lost (YLLs), and Healthy Life Expectancy (HALE) were of interest.

#### COI

Esther and Pancras [18] summarizes it as a term that quantifies the economic burden of a disease, including direct costs (medical expenses) and indirect costs (disability leave, work accidents), mainly reported as healthcare utilization. We included PL and psychosocial wellbeing as components of COI to consider relevant issues beyond economic factors, which include broader social and health impacts such as absenteeism, presenteeism, job loss, work performance decline, suicide risk, increased healthcare use, reduced-self-esteem, poor career prospects and workplace well-being. This comprehensive assessment captures both economic and human costs, highlighting the multidimensional impact of mobbing on individuals and society.

#### PL

PL refers to the reduction in an individual’s or workforce’s capacity to perform work-related tasks effectively due to health problems or adverse workplace conditions, encompassing both absenteeism (time away from work) and presenteeism (reduced performance while at work).

### Study Selection

**Fig. 1.**
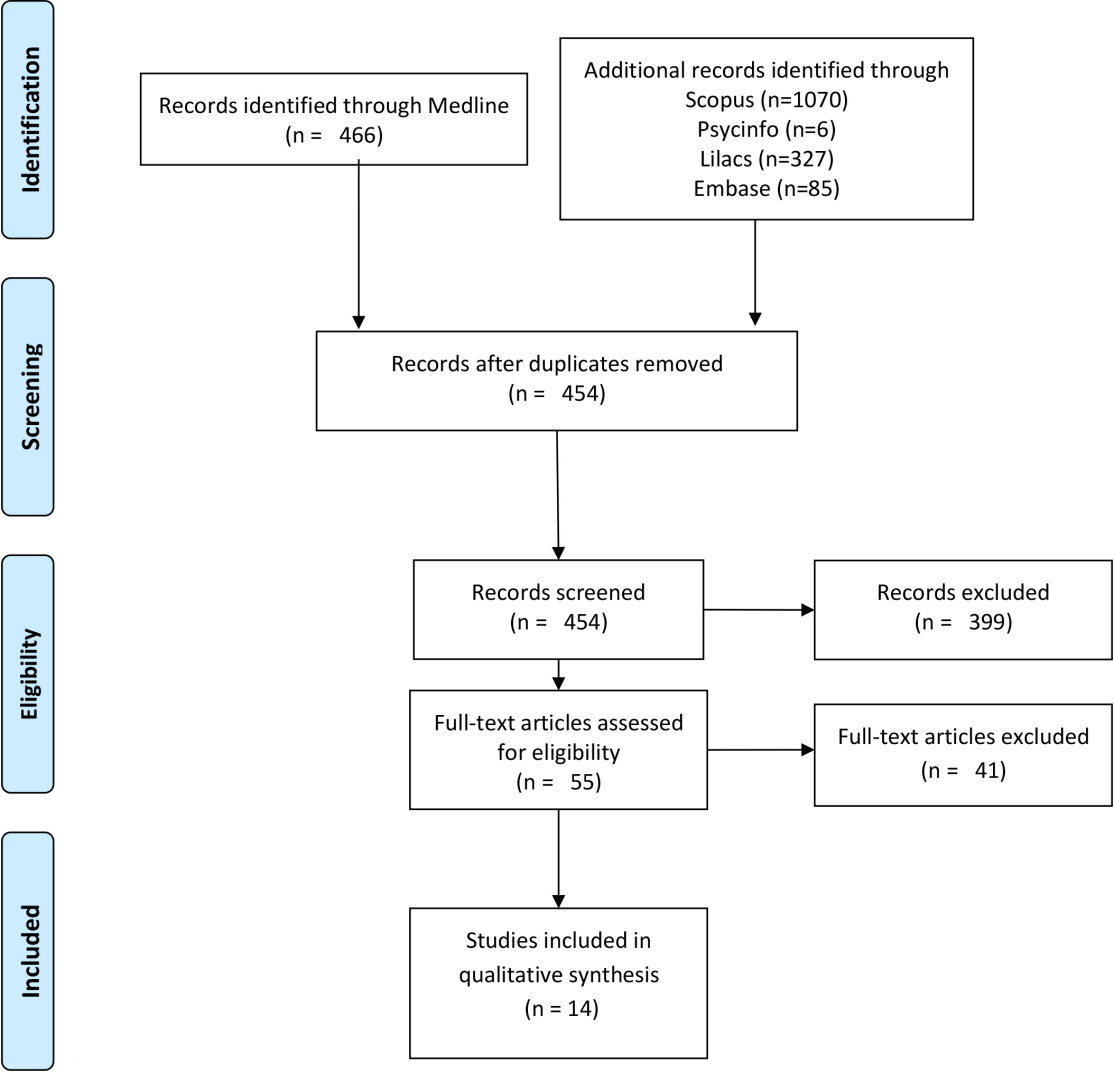
**Search strategy flowchart and results**.

Search results were reviewed for eligibility in two phases using the Rayyan web app for systematic and literature reviews [19]. First, title and abstract screening, followed by full-text review of preselected articles. This process was performed in duplicate by independent reviewers. Discrepancies were solved by consensus or by a third reviewer. Fig. 1 presents the selection process (PRISMA flowchart).

### Data Extraction and Synthesis

For each included study, the following variables were extracted: author, title, year, country, study design, population, objective, scales measuring exposures and outcomes, and main findings. The extracted data were summarized narratively according to five thematic domains: (1) mobbing and work environment characteristics; (2) Psychosocial factors; (3) COI; (4) Scales/measuring instruments; (5) Associations between mobbing and productivity.

## Results

The search strategy identified 454 articles. Of these, 55 were reviewed in full text, and 14 met the selection criteria and were included in the synthesis. The excluded articles did not mention mobbing or any of disease burden measures of interest (See Fig. 1).

The selected articles were published between 2008 and 2020 (Table 1, Ref. [20, 21, 22, 23, 24, 25, 26, 27, 28, 29, 30, 31, 32, 33]). Most were written in English (92.8%) and originated from Europe (50%), including two from Spain. The United States and Australia each contributed three articles, while one article came from Asia [20]. A total of 71.4% were original articles, most of them cross-sectional. Four articles were reviews (28.6%), including two systematic reviews [21, 22] and two narrative reviews [23, 24].

**Table 1.** **Main characteristics and results of the 14 studies included in the review**.

#	Author	Title	Country	Study design	Population	Objectives	Scale	Main findings
1	Roelen C.,* et al*. [29] (2018)	Psychosocial work environment and mental health-related long-term sickness absence among nurses.	Norway	Prospective 2 years.	Norwegian nurses, who work at least 50% of the time as a plant.	To investigate which work demands and resources were predictive of long-term mental health-related illness (LTSA) in nurses.	Harassment at the workplace: 9-item Negative Acts Questionnaire (NAQ-9)	After adjusting for demographic and job variables, harassment (OR = 1.06; 95% CI = 1.02–1.11) and social support (OR = 0.95; 95% CI = 0.91–0.99) remained significantly associated with LTSA. When these same variables were entered into another model with job demands and resources, they were shown to be strong predictors: harassment for all-cause LTSA, and social support for both all-cause LTSA and mental health-related absence.
2	Miller P.,* et al*. [26] (2020)	Bullying in Fly-In-Fly-Out employees in the Australian resources sector: A cross-sectional study.	Australia	Cross-sectional - Survey	Fly-in-fly-out (FIFO) workers from remote areas who are brought in to work temporarily instead of permanent relocation	Establish and identify the prevalence and predictors of mobbing. They also examine the relationship between bullying and depression and suicide risk. All Australian FIFO workers	The Negative Acts Questionnaire-Revised (NAQ-R).	Workers reported occasional workplace bullying (28.6%), while 27.1% experienced severe bullying. Older age reduced bullying likelihood by 50% (OR 0.51; 95% CI 0.31–0.83). Lack of supervisor collaboration tripled bullying risk (OR 3.04; 95% CI 1.84–5.04). Additionally, 32.3% reported moderate to severe depression, 26.7% had elevated suicide risk, and bullying nearly tripled suicide risk (OR 2.70; 95% CI 1.53–4.76).
3	Sabbath E.,* et al*. [30] (2018)	Mental Health Expenditures: Association With Workplace Incivility and Bullying Among Hospital Patient Care Workers.	USA	Cross-sectional	Hospital employees, with at least 20 working hours per week	To determine to what extent exposure to impoliteness and mobbing in the health environment is associated with a higher level of consultation for mental health services.	NAQ-R	Nearly a quarter of respondents reported being humiliated or ridiculed (21% incivility, 3% bullying), those exposed were slightly more likely to have mental health expenditures (*p* = 0.065); exposed workers were more likely to have mental health expenditures (*p* = 0.026). Being ignored or excluded was most strongly associated with mental health care use (OR = 2.51; 95% CI = 1.40-4.53), bullied workers had significantly higher expenditures (US 2461 versus unexposed workers (US 957, *p* = 0.003).
4	Nabe-Nielsen K.,* et al*. [32] (2016)	The role of poor sleep in the relation between workplace bullying/unwanted sexual attention and long-term sickness absence.	Denmark	Cohort	Employees in Denmark, public and private sector and the central bank of Denmark	To investigate whether sleep deprivation mediated the effects of and exposure to workplace bullying/sexual harassment on sickness absenteeism.	A subjective method of self-classification was used to say that she was bullied or not.	Exposure to bullying doubled the odds of LTSA (OR = 1.92; 95% CI = 1.63–2.30); 10.2% was mediated by disturbed sleep and 4.0% by difficulty waking up. Increased odds of LTSA among those exposed to unwanted sexual attention, 6.1% was mediated by disturbed sleep and 3.8% was mediated by difficulty waking up. Bullying increased the odds of sickness absence, disturbed sleep appears (OR = 1.23; 95% CI = 1.15–1.32) to be more important for this outcome than difficulties awakening (OR = 1.54; 95% CI = 1.06–2.24).
5	Hurley J.,* et al*. [25] (2016)	Nexus between preventive policy inadequacies, workplace bullying, and mental health: Qualitative findings from the experiences of Australian public sector employees.	Australia	Cross-section - Online survey - Open-response analysis	Public (primarily) and private (to a lesser extent) employees in a state in Australia	Make a qualitative description of the experience of exposure to harassment by public employees	Subjective self-reportage of experiencing bullying.	Emotional and psychological problems were common effects of workplace bullying. According to respondents, these included acute anxiety, depression, suicidal ideation, and suicide attempts. The emotional impact of bullying extended beyond the individual at work, affecting co-workers and family members. These spillover effects were described as significant, particularly for the family.
6	Hom M.,* et al*. [33] (2017)	Women Firefighters and Workplace Harassment: Associated Suicidality and Mental Health Sequelae.	USA	Cross-sectional	Female firefighters between 18 and 58 years old	1. Describe the prevalence of sexual harassment and other types of harassment in the workplace	The Quality of Worklife Module (QWM)	Individuals reporting a history of sexual harassment while on the job as a firefighter were significantly more likely than those without this history to report experiencing career suicidal ideation (Adjusted Odds Ratio [AOR] = 2.05; 95% CI = 1.12–3.74), controlling for pre-career ideation. Individuals who reported a history of other threats/harassment while on the job as a firefighter were significantly more likely than those without this history to report experiencing career suicide ideation (AOR = 2.42; 95% CI = 1.30–4.49) and career suicide plans (AOR = 2.80; 95% CI = 1.23–6.37), controlling for pre-career ideation and plans.
						2. Association between bullying and suicide		
						3. Association between bullying and psychiatric symptoms		
7	Figueiredo-Ferraz H.,* et al*. [31] (2012)	Influence of some psychosocial factors on mobbing and its consequences among employees working with people with intellectual disabilities.	Spain	Cross-sectional	Employees of Spanish companies who work with people with mental disabilities	To analyze the influence of role clarity, interpersonal conflicts and social support on mobbing and its consequences	Mobbing was evaluated with Mobbing-UNIPSICO scale, adapted from Leymann Inventory of Psychological Terrorization and the Negative Acts Questionnaire.	Mobbing correlated significantly and in the expected direction with psychosomatic disorders (r = 0.37, *p* < 0.001) and absenteeism (r = 0.18; *p* < 0.001). Psychosocial factors accounted for 37% of the variance in this study. The overall model explained 13.8% of psychosomatic disorders and 3% of absenteeism.
8	De Pedro M.,* et al*. [27] (2008)	Workplace mobbing and effects on workers’ health.	Spain	Cross-sectional	Agricultural employees in Murcia, Spain	To analyze the relationship between mobbing and psychosomatic symptomatology and absenteeism from work. Identify mobbing factors that predict psychosomatic symptoms	Negative Acts Questionnaire (NAQ-RE) which is a review of the Spanish adaptation of the NAQ-R.	A total of 102 employees indicated that they had taken leave (26.3%). Of this total, 32% were classified as victims, compared to 24% who were non-victims. No statistically significant differences were found between the groups (*p* = 0.123). The reasons for absenteeism were divided into two categories: those of a physical nature (work accidents, general physical complaints, flu, etc.) and those of a psychological nature (depression, anxiety attacks, headaches, etc.). The results showed no statistically significant differences, t(1) = 0.0639, α = 0.525.
9	Min J. Y.,* et al*. [20] (2014)	Workplace injustice and self-reported disease and absenteeism in South Korea.	South Korea	Cross-sectional	Korean economically active population, non-pensioners, non-unemployed, non-students, non-housewives, non-self-employed, and non-military	To explore whether the experience of injustice in the workplace is associated with occupational health problems	Korean Working Conditions Survey: Injustice at work was determined through subjective open-ended questions.	Workplace injustice was significantly associated with increased self-reported illness and absenteeism, even after adjusting for job-related factors. Prevalence rates were similar across genders; however, men had higher injury-related absences, while women had more health-related absences. Adjustments reduced prevalence ratios for all health outcomes, except for absenteeism due to accidents, which remained unaffected.
10	Leach L.,* et al*. [21] (2017)	Workplace bullying and the association with suicidal ideation/thoughts and behaviours: a systematic review.	Australia	Systematic Review (N = 337, including 12)	Articles in English from the PubMed, PsycINFO, Cochrane, SCOPUS and Web of Science (Core Collection) databases. Until June 2016, that refer to the association between bullying, workplace and suicide.	To summarize published studies reporting data on workplace bullying and suicidal ideation or behavior	Bullying was determined by the Leyman Inventory of Psychological Terror in 3 studies. 2 studies used the Negative Acts Questionnaire and another 2 used a checklist of bullying situations. The most common was self-reporting of bullying through open-ended subjective questioning.	Eight studies found a significant positive association between workplace bullying and suicidal ideation, and one study found a positive association with suicidal behavior. Bullied employees were twice as likely to report subsequent suicidal ideation (AOR 2.05; 95% CI = 1.08–3.89) after adjustment for age, sex, and job change. Four additional studies report data on the frequency of suicidal ideation among targets of workplace bullying. However, these studies do not provide information on whether there is an increased risk (or even an association) with suicidal ideation or behavior.
11	Lever I.,* et al*. [22] (2019)	Health consequences of bullying in the healthcare workplace: A systematic review.	United Kingdom	Systematic review (N = 8862, including 45)	Articles that discuss the consequences of bullying on the physical and mental health of health workers. From EMBASE, MEDLINE, PsycINFO, PUBMED and Web of Science Core Collection	Review the mental and physical health consequences of mobbing (bullying) in health employees.	It does not specify how the presence of bullying was measured within the included items.	The pool mean estimate of bullying prevalence was 26.3% (95% CI = 22.4–30.1). Forty of the 45 papers in the review examined mental health outcomes. Depression, burnout, psychological distress, anxiety, suicidal ideation or suicide attempts:
								Sick leave: Nine papers measured sick leave; seven showed significant associations with bullying and two did not comment on significance.
12	Johnson S. [23] (2009)	International perspectives on workplace bullying among nurses: a review.	USA	Narrative Review	Articles that evaluated workplace bullying in nursing, from the bases: CINAHL, PubMed, Pro Quest and EBSCO host.	Review the literature for a better understanding of workplace bullying in nurses	It does not specify how the presence of bullying was measured within the included items.	In a study of Turkish nurses, 10% of respondents said they had considered suicide because of workplace bullying. Victimization due to workplace bullying can ruin not only the mental health of employees, but also their careers, social status, and thus their way of life. Most studies of workplace bullying among nurses have found that nurses who have been bullied at work discuss leaving either their current job or the nursing profession as a result of their negative experiences.
13	Kobelt A.,* et al*. [28] (2010)	Do people with bullying experiences who apply for medical rehabilitation have a conspicuous personality? [Do people with mobbing experience which apply for medical rehabilitation have a peculiar personality?].	Germany	Cohort	Insured by the Braunschweig-Hannover Pension System who have been granted medical rehabilitation of some kind	Describe the relationship between mobbing, personality types, and anxiety and depression findings	Trierer Mobbing-Kurz-Skala (TMKS)	Among rehabilitation claimants, the average age was 48 years, the proportion of women was 42.3%, 53.6% claimed medical rehabilitation for physical problems, 8.7% for psychological problems and 37.7% for combined physical and psychological problems; 49.9% had been unable to work for more than 4 weeks in the previous 12 months and 24.2% had experienced harassment at work. Those who experienced harassment were more likely to present for treatment as incapacitated and had longer periods of incapacity in the year prior to their claim than those who did not experience harassment.
14	Bambi S.,* et al*. [24] (2018)	Workplace incivility, lateral violence and bullying among nurses. A review about their prevalence and related factores.	Italy	Narrative Review	Articles evaluating mobbing in the Medline, CINAHL, Embase base nurses	Detect the prevalence of workplace incivility, lateral violence, and harassment among nurses. In addition, to address the factors and their impact on the psychological and professional spheres of the victims.	Most of the articles mainly reported average values of specific scores as the Workplace Incivility Scale tool	Targets of bullying among nurses showed a deterioration in well-being (r = 0.40, *p* < 0.05), with bullying acting as a predictive factor for burnout (β = 0.37, *p* < 0.001). A negative correlation was also observed between bullying and work productivity (F = 0.045, r = –0.322, *p* < 0.01), and exposure to bullying was associated with a higher number of sick days than the average employee: 1.5 times (95% CI = 1.3–1.7) vs. 1.2 times 95% CI = 1.1–1.4), respectively.

NAQ-9, 9-item Negative Acts Questionnaire; LTSA, long-term sickness absence; FIFO, Fly-in-fly-out; OR, Odds ratio; NAQ-R, The Negative Acts Questionnaire-Revised; CI, Confidence interval; AOR, Adjusted Odds Ratio; TMKS, Trierer Bullying-Kurz Scale.

The number of participants ranged from 290 and 7650 (Table 1). Four studies had sample sizes exceeding 1000 participants. The included literature reviews summarized between 12 and 79 articles. Five studies focused on healthcare personnel, primarily nurses (four studies). The remaining articles examined populations from various sectors, including agriculture workers, firefighters and public employees.

### Mobbing and Related Occupational Factors

We did not identify any studies that directly quantified the BOD associated with mobbing using utility-based measures. All studies examined mobbing as a risk factor, including one qualitative study [25]. Six studies considered mobbing as the sole risk factor or predictor for various outcomes [21, 22, 23, 26, 27, 28], while another seven also measured additional factors such as job demands [29], incivility [24, 30], role ambiguity [31], interpersonal conflicts [31], social support at work [31], workplace injustice [20], lateral violence [24], and sexual harassment [32, 33].

### Intermediate Psychosocial Factors

In total, four articles examined intermediate psychosocial factors in the association between mobbing and outcomes related to the COI and PL [28, 29, 31, 32]. These factors included burnout, work stress, commitment and motivation at work, poor sleep quality and mental or other health issues. One study analyzed mobbing as an intermediate factor between other psychosocial factors and absenteeism.

### COI-Healthcare Utilization 

Healthcare utilization was assessed in two studies: Sabbath *et al*. [30] focused on mental health service use, examining expenses related to conditions such as anxiety, depression nonspecific neuroses, substance use, and eating disorders—conditions considered sensitive to environmental triggers. Total costs included inpatient, outpatient care (such as counseling and psychiatric consultations), as well as prescription medications, measured during the 12 months following the survey. The study found that workplace humiliation and ridicule raised mental health expenditures in hospital workers. Being ignored or excluded was the exposure most strongly associated with the use of mental health services with nurses incurring in significantly higher costs.

Kobelt* et al*. [28] investigated psychosomatic rehabilitation requests among workers affected by mobbing and reported that 24% of individuals who felt harassed had requested rehabilitation. Additionally, mobbing was associated with a 1.5-fold higher risk of occupational injuries [20, 23].

### COI-PL and Absenteeism

Absenteeism was the most frequently studied outcome (8 out of 14), defined as absence due to illness—any mental or behavioral International Classification of Diseases Tenth Revision (ICD-10) diagnosis—for 17 or more consecutive days, or 30 or more days in other cases [20, 22, 23, 24, 27, 29, 31, 32]. Three studies [20, 29, 32] reported a significant association between mobbing and absenteeism (OR between 1.06 and 1.92). Another study [31] found a significant but weak correlation between mobbing and absenteeism (r = 0.18; *p* < 0.001), while one study [27] reported no statistically significant difference regarding absenteeism between employees who had experienced mobbing and those who had not (*p* = 0.123).

Mobbing was associated with absenteeism rates 1.5–2 times higher, with increased healthcare costs due to increased mental health service use and long-term sickness absence (LTSA) [20, 29]. Similarly, Bambi* et al*. [24] reported that nurses exposed to mobbing had 1.5-fold higher absenteeism rates (95% CI: 1.3–1.7). Nabe-Nielsen *et al*. [32] concluded that sleep disturbances and difficulty waking in mobbing victims were associated with higher odds of absenteeism due to illness. Additionally, applicants for medical rehabilitation who reported being affected by mobbing experienced longer periods of work incapacity than those who were not affected [28].

### COI-Psychological and Social Wellbeing

The second most frequently studied outcome was suicide-related events (5 out of 14 studies), including suicidal ideation [21, 24, 28], suicidal behavior [28] and suicidal risk [26, 33]. Two studies also assessed the impact of mobbing on well-being [22], work productivity [22], loss of trust in the company/employer [25], abuse of power and traumatic emotional responses [25].

Hurley* et al*. [25] found that the emotional impact of mobbing extended beyond the individual, affecting co-workers and family members. These effects were described as significantly harmful, particularly to the family. Johnson [23] described how mobbing negatively affects nurses, ranging from suicidal ideation to diminished social status, changes in lifestyle, and damage to their professional careers, even leading some to consider leaving the profession.

Additionally, Lever *et al*. [22] reported that, for nursing staff, being a victim of mobbing was associated with an increased likelihood of committing errors with patients, which represents a significant risk for third parties and also contributes to the overall COI.

### Scales and Measuring Instruments 

Thirteen studies defined mobbing using terms such as harassment, bullying, workplace bullying, or workplace incivility. The Negative Acts Questionnaire (NAQ) was the most frequently used tool (35.7%), while others employed self-reported measures tailored to the specific study population [26, 27, 29, 30, 31]. The remainder used either their own self-report measures or instruments adapted to the study population or research designs.

Suicidal ideation or risk was assessed using tools such as the Beck Hopelessness Scale, the Acquired Capability for Suicide Scale and a revised 4-item Suicidal Behaviors Questionnaire [23, 28].

Regarding PL and COI, absenteeism was measured differently across studies. Figueiredo-Ferraz *et al*. [31] used the UNIPSICO subscale, while De Pedro *et al*. [27] and Min *et al*. [20] defined absenteeism through direct questions about recent work-related absences. The first used a closed-ended question (“Were you absent in the last six months?”) along with an open-ended question about the reason for the absence. The latter assessed work-related absenteeism with the questions “in the previous twelve months, have you been absent from work for more than one day due to a work-related accident?” and “In the previous twelve months, have you been absent from work for more than one day due to health problems caused by work?”.

## Discussion

### Summary of Findings

This review mapped and summarized the literature assessing the BOD associated with mobbing, focusing on the deterioration of health, COI and PL. Although no studies were found that quantified the BOD of mobbing using utility-based measures, we identified a substantial body of evidence documenting the harmful consequences on COI, particularly its impact on increased absenteeism, PL, healthcare utilization and various consequences on victims’ wellbeing.

### Populations Studied

Health personnel were the most common population group among the studies included. There is a well-documented history of mobbing in the healthcare field, dating back to undergraduate training [34, 35, 36, 37]. This suggests that such relational dynamics are learned early and sustained throughout professional environments, as reflected in reports of suicide, career abandonment, prolonged or permanent mental disability and other negative outcomes. The hierarchical and high-pressure nature of healthcare settings may inadvertently create conditions that facilitate mobbing. Several studies highlight that mobbing can exacerbate the current shortage of healthcare professionals by decreasing job satisfaction and organizational commitment, while increasing the likelihood of leaving the profession [23, 38]

### BOD

Assessing the burden of mental disorders presents challenges, as disability from mental illness is often measured as loss of health, excluding aspects of well-being. This approach may underestimate the overall impact on individuals and society [5]. Additionally, traditional burden-of-disease models assume independent distributions of comorbidities a condition which may not apply to mental disorders and their systemic consequences, further complicating estimations [5].

Studies of the burden of mental illness such as those by Soriano* et al*. [39] and Dantes Gomez *et al*. [40], have reported multiple pathologies, including anxiety and depression, in terms of DALYs and QALYs, for the population of Spain and Latin America respectively.

### PL 

Absenteeism has been widely studied as an indicator of PL affecting all stakeholders involved in the workplace, including employers, employees and insurers, etc. In this review, it was the most frequently reported outcome. However, its definition is not standardized. The use of different methods to assess PL, including scales that use “motivation to work” as an indicator and open-ended questions to determine the reason for the absence is noteworthy and should inform future research on the subject.

Regarding the association between mobbing and absenteeism, studies that identified a significant association had larger sample sizes (ranging from 1500 to 7000 compared to approximately 500) [20, 31, 32]. Additionally, it is estimated that reducing mobbing may result in cost savings by decreasing sick leave and mitigating costly events associated with presenteeism [22].

### Loss of Wellbeing

Another factor contributing to the COI associated with mobbing was suicide and suicide-related events, which was examined in one-third of the studies [21, 24, 28, 33]. The general literature indicated that mobbing increases the risk of suicide approximately threefold (OR = 2.70; 95% CI = 1.53–4.76), a phenomenon explained by the interpersonal theory of suicide, which is based on two key pillars: the feeling of social alienation and the perception of being a burden. The implications of a worker’s suicide in terms of lost productivity extend beyond the direct loss of the individual, their experience and their knowledge; it also has profound effects on co-workers, sometimes with severe and far-reaching consequences [41, 42, 43].

The consequences of mobbing not only affect victims, but also have significant economic implications for employers, who face increased healthcare costs and reduced organizational efficiency. Despite the relevance of this problem for both public health and workplace environments, we identified only one study that specifically addressed it. This study found an association between greater use of mental health services and higher health care expenditures among individuals exposed to workplace incivility or mobbing [30, 44].

### Mobbing in Healthcare Workers

The prevalence of mobbing among healthcare workers is a critical issue driven by several factors. Firstly, the healthcare sector exhibits a notably high prevalence of mobbing behaviors [45, 46, 47]. Secondly, mobbing has profound implications for the well-being of healthcare professionals. Exposure to such workplace mobbing is associated with both physical and mental health deterioration, significantly impacting their quality of life. Since healthcare workers are essential to patient care and overall public health, understanding and mitigating these negative outcomes is crucial [48].

The high risk identified among health care workers does not imply that other populations are unaffected. However, there may be less accessibility or interest in conducting studies on other groups. In this review, we identified one study on public employees, another on firefighters, and one on agricultural sector workers. This finding aligns with the broader literature, which also highlights the education sector as another at-risk group.

### Mobbing and Other Exposure Factors

Half of the studies evaluated mobbing alongside other protective or risk factors [20, 24, 29, 30, 31]. Mobbing has been described as a form of violence that is highly detrimental to both mental and physical health, inducing fear, terror, shame and other emotions, taht ultimately undermine self-esteem, leading to anxiety, sadness, loneliness, isolation and self-stigmatization.

Several studies highlight that mobbing and workplace injustice are associated with an increased risk of occupational disease and absenteeism in both men and women. The effect can be even greater when mobbing interacts with other well-established toxic factors known to induce burnout and mental illness, thereby contributing to PL. Additionally, this relationship may be influenced by external pressures from company managers or co-workers, particularly in environments where there is a high risk of job loss or when victims fear being accused of feigning illness.

The stress response related to mobbing is a subject of growing interest. Roelen *et al*. [29], suggested that mobbing and other psychosocial factors may play either a protective or risk enhancing role in the exacerbation of mental disorders. Organizational characteristics and workplace dynamics, such as role clarity or ambiguity, fair or unfair leadership, lack of recognition, and the presence of hostile interpersonal environments appear to be key elements in the process.

The importance of this topic lies in its potential to inform strategies for preventing and mitigatigating the effects of mobbing on disability processes and return-to-work outcomes [49]. A complementary approach suggests that, in addition to organizational interventions, individual should also be encouraged to take greater self-responsibility in enhancing their resilience to bullying [25].

### Mobbing Measurement Scales

The most used instrument across the reviewed articles was the NAQ. The NAQ has been frequently employed to evaluate mobbing, with psychometric validation in specific populations. Notable examples include the study by Escartín *et al*. [50] which asssessed a version designed for perpetuators, and the study by Millán de Lange *et al*. [51] which validated a version for use among Venezuelan workers.

The NAQ is a robust and widely recognized tool for measuring workplace bullying. However, it should be complemented with other qualitative or context sensitive methods to provide a more comprehensive and accurate assessment of the mobbing phenomenon.

### Limitations

We identified a significant challenge in synthesizing information due to the ambiguity and lack of standardization in terminology, as well as the variety of tools and scales used to assess mobbing in the included studies. In recent years, there has been a noticeable increase in publications addressing mobbing. This allowed for a demographic characterization of the factors identified, as well as an analysis of existing knowledge gaps. However, although mobbing has gained greater recognition within the scientific community, it reamins unclear whether this is due to an actual increase in harassment incidents or a rise in reporting. This underscores the need for a standardized approach to defining mobbing and measuring its consequences.

While we identified studies from most continents, more than half were conducted in Europe. This trend is likely influenced by higher economic and social development in European countries, as well as a stronger emphasis on assessing social issues to inform legislative measures related to occupational mental problems. Other regions of the world are likely underrepresented, and the nature and magnitude of mobbing’s consequences may differ across contexts.

Self-reported questionnaires in mobbing research have inherent limitations. One major concern is social desirability bias, where respondents may underreport or overreport their experiences due to perceived social norms or fear of stigma [10]. Recall bias is another issue, as participants may inaccurately remember or misinterpret past experiences, particularly when assessing long-term effects such as mental health outcomes [10]. Additionally, variability in the definition and perception of mobbing, reflected in the diverse measurement tools used, ranging from standardized instruments like the NAQ to subjective, open-ended questions [10, 52], further complicates direct comparisons across studies. This methodological heterogeneity reduces the reliability of prevalence estimates [10, 21, 22].

### Policies and Workplace Suggestions

To address the burden of mobbing and mitigate its negative effects, it is important to implement comprehensive, multilevel policies that promote a safe and supportive work environment [53]. Establishing and enforcing clear anti-mobbing policies—defining workplace harassment, outlining consequences for perpetrators, and confidential reporting mechanisms—can empower victims to come forward without fear of retaliation [54]. Beyond policy implementation, fostering a positive organizational culture is equally critical [55]. Leadership development programs, conflict resolution training, and team-building initiatives can improve workplace dynamics and reduce interpersonal tensions, while providing mental health resources and support networks can offer employees guidance. Additionally, flexible work arrangements or temporary accommodations for affected employees can facilitate recovery and help prevent work disability and return to work challenges [49]. To strengthen these efforts, conducting anonymous surveys to assess the workplace climate can help organizations identify and address mobbing before it escalates.

Furthermore, effective management mobbing requires broader public health and legislative actions, including clear legal protections for victims and mandatory reporting of incidents to enhance accountability and prevention [56]. Given its substantial economic burden—including increased absenteeism, presenteeism, and healthcare costs—organizations should view the prevention of mobbing not only as an ethical obligation but also as a sound investment. Workplace wellness programs, when properly implemented, have shown positive outcomes in reducing stress-related conditions and improving productivity [57]. Additionally, the healthcare sector plays a key role by training professionals to recognize and address mobbing-related health issues [58], while integrating workplace stress factors into mental health care. This integrative approach can ensure more comprehensive care for affected individuals.

### Future Directions

Two issues that warrant further evaluation are the use and abuse of psychoactive substances and the relationship between mobbing and sexual harassment. These areas of study should be expanded, considering the potentially high emotional toll in cases of sexual harassment, and the pathophysiological implications associated with psychoactive substance use.

Future research should also aim to quantify the BOD caused by mobbing in terms of its impact on quality of life, including the use utility-based measures. Given the wide range of consequences associated with mobbing, such as mental health disorders and diminished wellbeing, significant effects in health-related quality of life and utility measures would be expected.

As anticipated, important knowledge gaps remain. These include the lack of longitudinal studies that can establish clear causal relationships, and the need for more precise estimates of the overall economic burden. Moreover, the definitions of mobbing and the measurement of its severity required standardization to improve comparability across studies.

## Conclusions

Workplace mobbing is a topic of growing interest, with significant relevance for workplace stakeholders and for mental health professionals. Its consequences extend across various domains, affecting mental and overall health, and society wellbeing, making it a phenomenon of interest for public health.

Although stigma hinders timely detection and intervention, awareness among both employees and employers is essential for prevention. Additionally, the increase demand for health care services resulting from mobbing should raise economic concerns. Despite its recognized impact and burden, research on its associated costs and effects on quality of life remains scarce. There is also a need for standardized definitions and measurements of severity.

## Availability of Data and Materials

All data generated or analyzed during this study are included in this published article.
